# Long-Term Regional Shifts in Plant Community Composition Are Largely Explained by Local Deer Impact Experiments

**DOI:** 10.1371/journal.pone.0115843

**Published:** 2014-12-31

**Authors:** Katie Frerker, Autumn Sabo, Donald Waller

**Affiliations:** 1 Department of Botany, University of Wisconsin, Madison, Wisconsin, United States of America; 2 Department of Forest and Wildlife Ecology, University of Wisconsin, Madison, Wisconsin, United States of America; USDA-ARS, United States of America

## Abstract

The fact that herbivores and predators exert top-down effects to alter community composition and dynamics at lower trophic levels is no longer controversial, yet we still lack evidence of the full nature, extent, and longer-term effects of these impacts. Here, we use results from a set of replicated experiments on the local impacts of white-tailed deer to evaluate the extent to which such impacts could account for half-century shifts in forest plant communities across the upper Midwest, USA. We measured species' responses to deer at four sites using 10–20 year-old deer exclosures. Among common species, eight were more abundant outside the exclosures, seven were commoner inside, and 16 had similar abundances in- and outside. Deer herbivory greatly increased the abundance of ferns and graminoids and doubled the abundance of exotic plants. In contrast, deer greatly reduced tree regeneration, shrub cover (100–200 fold in two species), plant height, plant reproduction, and the abundance of forbs. None of 36 focal species increased in reproduction or grew taller in the presence of deer, contrary to expectations. We compared these results to data on 50-year regional shifts in species abundances across 62 sites. The effects of herbivory by white-tailed deer accurately account for many of the long-term regional shifts observed in species' abundances (R^2^ = 0.41). These results support the conjecture that deer impacts have driven many of the regional shifts in forest understory cover and composition observed in recent decades. Our ability to link results from shorter-term, local experiments to regional long-term studies of ecological change strengthens the inferences we can draw from both approaches.

## Introduction

Ecological processes occur and interact at various spatial and temporal scales [1]. Most ecological research occurs over short periods of time and is limited in its spatial extent, restricting our understanding of longer-term and broader-scale processes [2]. The resulting gaps in our knowledge are sometimes labeled the “invisible present” [3] and the “invisible place” [4]. Without baseline data to provide a reference, it is difficult to infer what long-term changes may be occurring and whether short-term local studies are representative or merely anecdotal [5]. Although top-down effects of particular herbivores and carnivores are now well-recognized in the literature, these results have generally been demonstrated to occur locally and over short periods of time. Importantly, we now have ample evidence that deer can dramatically affect plant communities derived from fenced exclosure studies, island studies, and direct observation. Nevertheless, doubts and uncertainty persist concerning whether such impacts are serious or pervasive and how long they persist [6], [7], [8], [9], [10], [11].

It has proved surprisingly difficult to understand how factors affecting local populations accumulate into regional meta-population dynamics, particularly when the ecological forces affecting those populations vary conspicuously over time and space. The complexity of these dynamics makes it difficult to disentangle the impacts of deer from other drivers and to predict how plant communities may respond cumulatively to deer over long time periods and broad spatial scales.

Long-term “then vs. now” studies replicated over many sites reveal that forest plant communities in northern Wisconsin have undergone significant shifts in composition and diversity since the 1950s [31], [32]. These changes include declines in mean alpha (site) diversity, floristic quality, and community homogenization (declines in beta diversity among sites) as well as conspicuous increases in some species and declines in others [33]. These observed long-term regional changes in community composition could reflect the action of many ecological factors including succession, habitat fragmentation, climate change, and aerial nitrogen deposition as well as deer impacts.

Deer of several species have increased in abundance across much of North America in recent decades [12], [13]. In the Upper Midwest, USA, increases in white-tailed deer (*Odocoileus virginianus*) are thought to reflect favorable habitat conditions (landscapes with mixtures of forest, openings, and agricultural fields), scarce predators, limits on hunting (especially does), and, in northern parts of their range, mild winters and supplemental winter feeding [12], [14], [15]. At current densities, deer threaten tree regeneration [16], [17], [18], [19], [20], [21] and the growth and diversity of many understory species [22], [23], [24], [25]. Failures in tree regeneration incur serious economic impacts. The decline and loss of plant cover, structure and diversity further threaten ecosystem health and function [12]. These impacts of white-tailed deer reach beyond direct effects on vegetation to include many indirect effects on forest birds, mammals, and invertebrates [26], [27], [28], [29], [30]. The impacts of ungulate browsing are not restricted to one region or deer species. Researchers around the globe are assessing deer species' impacts to vegetation and their role in trophic systems, finding detrimental effects that permeate through several ecosystem levels [62], [63]. In sum, deer are acting as keystone herbivores [15], [26] in many regions around the world, affecting community structure and many ecosystem processes.

Deer impacts on woody plants are often inferred from demographic profiles or remnant stems. Assessing deer impacts on understory species presents more challenges as herbaceous species leave few traces of previous herbivory or may disappear altogether. Researchers instead document deer impacts on herbaceous species by observing reductions in abundance, height and/or the number of reproductive individuals [34], [35], [36], [37]. Alternatively, researchers can use fenced exclosures to directly compare and evaluate the effects of deer (or other mammals) on plant species over time. Exclosure studies can be highly informative, particularly if they are replicated across landscapes and regularly maintained. However, they can also exaggerate estimates of deer impacts relative to less extreme comparisons or underestimate deer impacts if plant communities were already impoverished prior to the time they were erected [38], [39]. Recovery within exclosures is often manifested by a gradual increase in height and recruitment. This recovery, however, may be limited if the less palatable or browse-resistant plants already present create a “recalcitrant understory” that prevents recolonization by browse-sensitive species [9], [40], [41], [42]. The slow growth of plants under shady conditions can also prevent exclosures from providing accurate estimates of impacts or recovery, particularly over shorter time periods and at sites lacking light gaps [43], [44]. Finally, when deer browsing preferences vary spatially, the impacts we infer from any one exclosure may lack generality. In sum, we are often uncertain of the generality of the inferences we can draw from smaller-scale exclosure studies even when these demonstrate locally dramatic impacts.

Others use ‘natural experiments’ to compare changes in community composition among sites or regions with different deer densities and presumed impacts [62]. Resurveys of sites with accurate baseline data are scarce but highly informative [45], [46], [47]. Although such studies often expose substantial ecological changes, they do not reveal their causes. In northern Wisconsin, Rooney et al. [32] noted that plant diversity declined the most in three state parks while diversity has not declined in three Indian reservations. These observations and the trends regarding which species increased and decreased led Wiegmann and Waller [33] to hypothesize that deer may account for many of the changes observed over the past half-century in Wisconsin's northern forests. These changes include declines in animal-pollinated native forbs and increases in wind-pollinated sedges and grasses. The extent to which such trends reflect the effects of deer herbivory [48], [49], [50], [51] or some other factor, however, remains unknown.

Here, we sought to isolate and identify the particular effects of deer on community change using a set of regionally distributed, long-term fenced exclosures. Comparing matched samples in- and outside the fences at these study sites allowed us to rigorously isolate the effects of deer from other factors known to affect plant community composition and dynamics. To increase the generality of the conclusions we could draw, we sampled 17 exclosures across seven sites. We then compared results from these sites to the patterns that emerged from a previous regional assessment of plant community change between the 1950s and 2000 based on quantitative resurveys of almost 10,000 quadrats sampling the forest understory at 62 sites [32], [33]. This allowed us to assess the degree to which deer herbivory has driven regional long-term changes and to demonstrate the power of explicitly linking complementary results from distinct studies. Such combined approaches are inherently more powerful and reliable than studies that focus on the effect of a single factor at one place and time [15], [20], [38], [52].

## Materials and Methods

### Study areas

**Figure 1:**
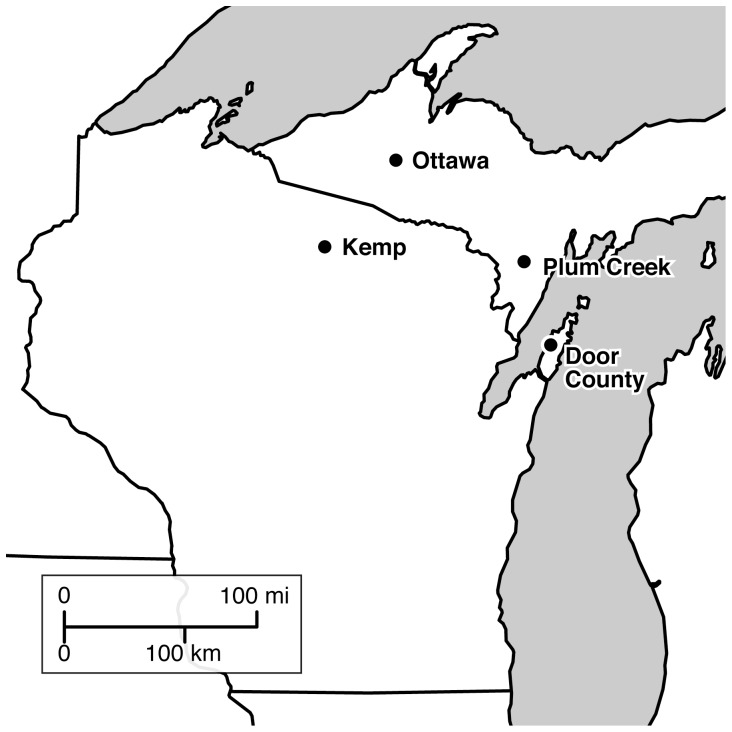
Map of study sites. Exclosure and browsed plots are located in Northern Wisconsin and the Upper Peninsula of Michigan in forests dominated by sugar maple with a hemlock component.

We used 17 experimental exclosures distributed across seven study sites in northeastern and north-central Wisconsin (WI) and the western portion of Michigan's (MI) Upper Peninsula (UP) (Fig. 1). Field research was conducted at seven sites, falling under the jurisdiction of four entities. Permission to work at each location was granted by:

Plum Creek Timber Company site: Gary Wyckoff, Region Silviculturist, Plum Creek Timber Company, 2831 North Lincoln Road, Escanaba, MI 49829-9569, Phone 906-789-9076 ext. 12, FAX 906-789-9130. Ottawa National Forest sites: Dave Steffensen, Silviculturist, R9 FACTS/TIM GIS Coordinator, Bergland, Kenton, and Ontonagon Ranger Districts - Ottawa National Forest, 1209 Rockland Rd, Ontonagon, MI 49953, Phone: 906-884-2411 ext. 29, Email: dsteffensen@fs.fed.us. Kemp Natural Resources Station site: Thomas W. Steele, Kemp Natural Resources Station, 9161 Kemp Road, Woodruff, WI 54568, phone: 715-356-9070, kemp@cals.wisc.edu. Door County State Parks sites: Kathleen Harris, Peninsula State Park, 9462 Shore Road, Fish Creek, WI 54212, phone: 920-868-3258, email: kathleen.harris@wisconsin.gov.

All exclosures were located in northern upland forests dominated by *Acer saccharum* (sugar maple) with some *Tsuga canadensis* (hemlock). The three northeastern WI sites lay in Door County on a limestone peninsula that extends into Lake Michigan. The exclosures are located in three state parks in forests dominated by *A. saccharum*, *Fraxinus* spp. (ash), *Populus* spp. (aspen), and *Betula papyrifera* (paper birch). The north-central WI site, Kemp Natural Resources Station (Kemp), lies alongside Tomahawk Lake in a mixed forest of northern hardwoods and second-growth hemlock. Dominant species include *A. balsamea*, *A. saccharum*, *Betula* spp. (birch), and *T. canadensis*. Two other sites lie in the Ottawa National Forest, just south of Kenton, MI with exclosures in forests dominated by *A. saccharum*, *Betula allegheniensis* (yellow birch), and *T. canadensis*. The final study site, Plum Creek, lies 25 miles west of Escanaba, MI on land owned by the Plum Creek Timber Company. Canopy dominants include *A. saccharum*, *Fraxinus pennsylvanica* (green ash), and *Ostrya virginiana* (hophornbeam).

### Exclosures and sampling

The fenced exclosures vary in age, size, and design reflecting their different owners and histories. We therefore varied plot placement and sampling to match the shape and area of each exclosure. For each plot, we also identified and sampled an adjacent control (browsed) plot matched for soil, management history, and overstory conditions. Within each plot, we surveyed multiple 1 m^2^ quadrats evenly spaced 2.5–5 m apart along successive parallel transect lines. Across the three sites in Door County, WI, we used four medium-sized circular exclosures constructed in 1991 and 1992 that measured 15.2 m in diameter (182 m^2^). In each, we sampled 21 quadrats arranged along 5 transects spaced 2.5 m apart. At the Kemp site, we sampled 16 quadrats in each of ten square exclosures (100 m^2^) constructed in 2001. At the two Ottawa sites, we sampled 33 quadrats in each of two plots within each of the large (2 ha) exclosures erected in 1998 and 2002. At the Plum Creek site, we again sampled 33 quadrats in each of two plots within the one large (8 ha) exclosure constructed in 1996. Together, these provided 20 exclosure plots, paired with 20 adjacent control plots, with 16–33 quadrat samples for each. For more details on sampling, see Frerker [53].

Within each quadrat, we recorded the incidence of all herbaceous, shrub, and tree seedling species. Where species could not be reliably distinguished, we later lumped species to genera (e.g., *Carex* and *Viola*). We estimated the abundance of each taxon by summing its incidence across all quadrats in the plot. For shrubs, we also recorded the total length (cm) that each shrub species' foliage intercepted our transect lines. We sampled the exclosure and control plots within 36 hours at each site. All sites were surveyed between June and August, 2011.

To assess whether deer particularly affect those species known to have increased or decreased across the region over the past 50 years (the “Winners” and “Losers” identified by Wiegmann and Waller [33]), we analyzed exclosure effects on these species separately. Thirty four of these 42 species occurred in one or more of the exclosure plots including 17 Winners and 17 Losers (Table 1). When these species were present in a quadrat, we scored the number of individuals browsed, how many were reproductive, and their maximum leaf height within the quadrat. We also collected these data on two other species (*Trillium grandiflorum* and *Uvularia grandiflora*) known to have declined and selectively favored by deer [34], [35], [54], [55]. We term the 34 increasing and decreasing species present in the plots plus these two others “focal” species.

**Table 1:** Species that significantly increased (“Winners”) or decreased (“Losers”) in abundance over the past 50+ years in the study area [33].

Winners	Losers
*Anemone quinquefolia*	*Aralia nudicaulis*
*Arisaema triphyllum*	*Circea alpina*
*Athyrium filix-femina*	*Clintonia borealis*
*Carex* spp.	*Cornus canadensis*
*Cinna latifolia*	*Diervilla lonicera*
*Corylus cornuta*	*Eurybia macrophylla*
*Dryopteris intermedia*	*Fragaria virginiana*
*Galeopsis tetrahit*	*Galium aparine*
*Hieracium aurantiacum*	*Huperzia lucidula*
*Maianthemum canadense*	*Mitchella repens*
*Oryzopsis asperifolia*	*Pteridium aquilinum*
*Poa nemoralis*	*Rubus parviflorus*
*Poa saltuensis*	*Streptopus lanceolatus*
*Trientalis borealis*	*Trillium grandiflorum* *
*Vaccinium angustifolium*	*Uvularia grandiflora* *
*Veronica officinalis*	*Uvularia sessilifolia*
	*Viola* spp.
	*Waldsteinia fragarioides*

*enumerated in other studies - see text.

### Data on 50-year shifts in abundance

To test whether the differences in species abundance that we observed in- vs. outside the exclosures were related to the long-term (1950s to 2001) regional shifts in abundance measured by Wiegmann and Waller [33] over 62 sites, we first calculated the proportional change in abundance across the fence line as the log of each taxon's frequency in the control browsed plot divided by its frequency inside the exclosure. We then calculated each taxon's regional proportional change in abundance over the last 50 years as the log of its total abundance at all 62 sites in 2000 divided by its abundance in the 1950s. We did this for all 20 species that occurred at reasonable density (5+ times) in both data sets. This represents a representative sample of the metacommunity species pool biased toward the more common species. We then plotted these estimates of regional long-term changes in abundance as a function of the average local, short-term shifts in abundance across the fence line. Because rare species are more likely to have undergone regional declines over the late 20^th^ century than common species [32] and are more likely to be palatable to deer [33], this approach underestimates actual deer impacts.

### Data analyses

To determine how the exclosure treatment affected species' abundances, we analyzed effects of the fence at different spatial scales. To test whether study area affected patterns of species occurrence, we applied logistic regression to assess how each species' presence/absence at a site varied over the seven study sites (3 Door County, 1 Kemp, 2 Ottawa, 1 Plum Creek) and two exclosure treatments (in or out). To assess how deer herbivory has affected forest structural diversity (the abundance of shrub species), we used mixed model ANOVA to test how each shrub species' abundance (log-transformed line intercept total) was affected by excluding deer (a fixed effect) and site (Door County, Kemp, Ottawa, Plum Creek, a random effect). We omitted four plots at Kemp where no shrubs occurred. Finally, to assess the overall regional effects of deer, we pooled data across all four sites and 20 paired plots to assess how each species responded to the presence of deer using Chi-squared tests. We restricted these tests to species that occurred in at least 50 quadrats overall and had expected frequencies greater than five in each treatment.

We similarly analyzed reproductive condition and plant height in the focal species (Table 2). We compared counts of the frequency of reproductive individuals in the protected and unprotected quadrats using χ^2^ tests. We compared measures of mean maximum leaf height in these focal species using a mixed model ANOVA with exclosure as the main effect and site as a random effect. To assess how deer affect species invasions, we used a 2×2 χ^2^ test to compare the ratios of native to exotic species between treatments. We performed all analyses using R 2.11.0 (R Development Core Team 2010) and JMP (Vers. 9.0).

**Table 2:** Differences in species abundances in- and outside deer exclosures.

	Abundance		
Species	Control	Exclosure	Chi-square value
**More Abundant in Control:**			
*Carex pensylvanica*	49	11	24.07 ***
*Ostrya virginiana*	41	25	3.88 *
*Lapsana communis*	61	14	29.45 ***
*Poa pratensis*	49	29	5.13 *
*Athyrium filix-femina*	59	25	13.76 ***
*Oryzopsis asperifolia*	57	33	6.40 *
*Prunus virginiana*	62	30	11.13 ***
*Anemone quinquefolia*	72	48	4.80 *
*Galium* spp.	53	23	11.84 ***
*Poaceae* spp.	213	109	33.59 ***
*Fern* spp.	236	169	11.08 ***
**More Abundant in Exclosure:**			
*Mitchella repens*	14	36	9.68 **
*Diervilla lonicera*	17	34	5.67 *
*Thuja occidentalis*	5	54	4.07 ***
*Trillium grandiflorum*	27	51	7.38 **
*Tsuga canadensis*	28	52	7.20 **
*Acer rubrum*	35	56	4.84 *
*Rubus idaeus*	77	116	7.88 ***
*Rubus* spp.	111	167	11.28 ***

Abundance values reflect the incidence of species across quadrats in the control and exclosure plots. We judge whether species are more abundant in or outside exclosures from the χ^2^ and significance values for the differences observed. No significant differences in abundance were observed in: *Acer saccharum*, *Dryopteris intermedia*, *Prunus serotina*, *Anemone americana*, *Eurybia macrophyyla*, *Taraxacum officinale*, *Aralia nudicaulis*, *Fraxinus pensylvanica*, *Tilia americana*, *Arisaema triphyllum*, *Maianthemum canadense*, or *Trientalis borealis*. ***p<0.001, **p<0.01, *p<0.05.

## Results

### Species' responses to deer

Of the 256 species encountered in the exclosure and browsed plots, 31 occurred commonly enough (a total frequency >50) to analyze differences in abundance in- and outside the fences using χ^2^ tests. Eight species were more abundant in browsed plots while seven were more abundant in the exclosures (Table 2). The other 16 species did not differ in abundance between treatments. Species that increased in the presence of deer included a forb (*Anemone quinquefolia* – 1.5×), a fern (*Athyrium filix-femina –* 2.4×), three graminoids (*Oryzopsis asperifolia –* 1.7×, *Carex pensylvanica* – 4×, and *Poa pratensis* – 1.7×), and two woody species (*Ostrya virginiana* – 1.6× and *Prunus virginiana* – 2.1×). Exotic species also increased being almost twice as abundant in the browsed plots vs. the exclosures (424 vs. 221 occurrences, χ^2^ = 61.2, p<0.0001). Conversely, several woody species, *Rubus* spp., and two forbs (*Mitchella repens* and *Trillium grandiflorum*) declined. Seedlings of *Thuja occidentalis* and *Tsuga canadensis* were much scarcer outside the exclosures. The shrubs *Cornus rugosa* and *Diervilla lonicera* also occurred 100–200 times more often within the exclosures than in the browsed plots (mean intercepts of 328.3 vs. 15.5 cm and 199.7 vs. 2.0 cm, respectively, Table 3). Responses of *Rubus allegheniensis* also varied over sites reflecting geographic variation in browse impacts. Combining taxa to higher levels, *Galium* spp., ferns, and grasses were all more abundant in the browsed plots. In sum, graminoids, ferns, and exotic species all thrived in the presence of deer while forbs, several shrubs and tree seedlings tended to decline.

**Table 3:** Results from ANOVAs of shrub abundance.

Species	F	d.f.	P-value
*Cornus rugosa*	6.2	7, 26	0.0002
Site	9.8	3	0.0002
Exclosure	2.9	1	0.10
Interaction	3.6	3	0.028
*Diervilla lonicera*	3.8	7, 26	0.006
Site	3.9	3	0.020
Exclosure	13.3	1	0.001
Interaction	1.1	3	0.36
*Rubus allegheniensis*	3.8	7, 26	0.006
Site	2.6	3	0.07
Exclosure	0.6	1	0.43
Interaction	6.21	3	0.0025

The Table shows F-values for predictor variables of shrub-line intercept values (log-transformed) with site, exclosure and their interaction as factors.

### Focal species responses

**Figure 2:**
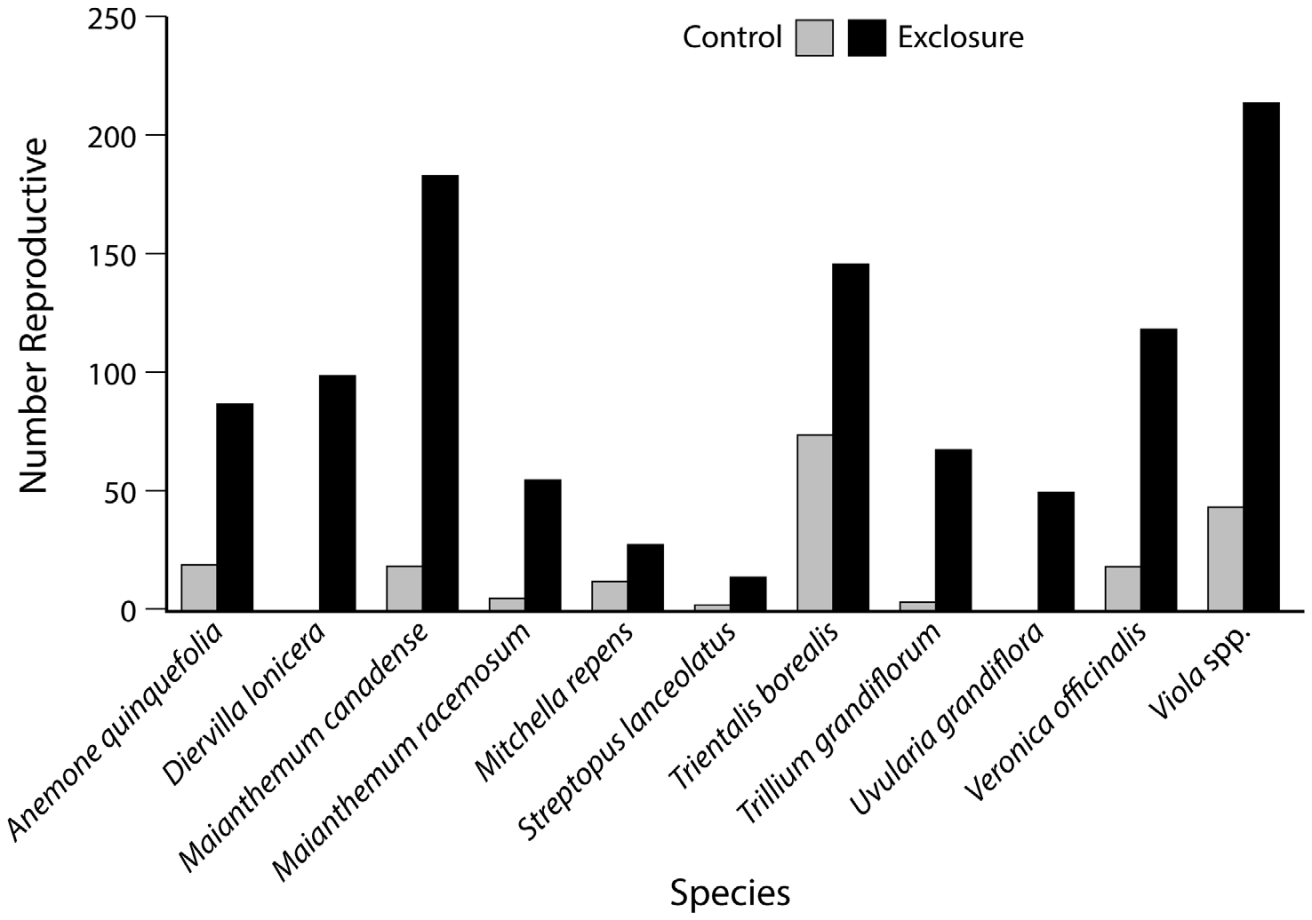
Effects of deer on plant reproduction. Bars compare the total number of reproductive individuals encountered for 11 focal species between the exclosure (protected from deer) and the control plots (accessible to deer). These species differ significantly between treatments in the χ^2^ analyses.

**Figure 3:**
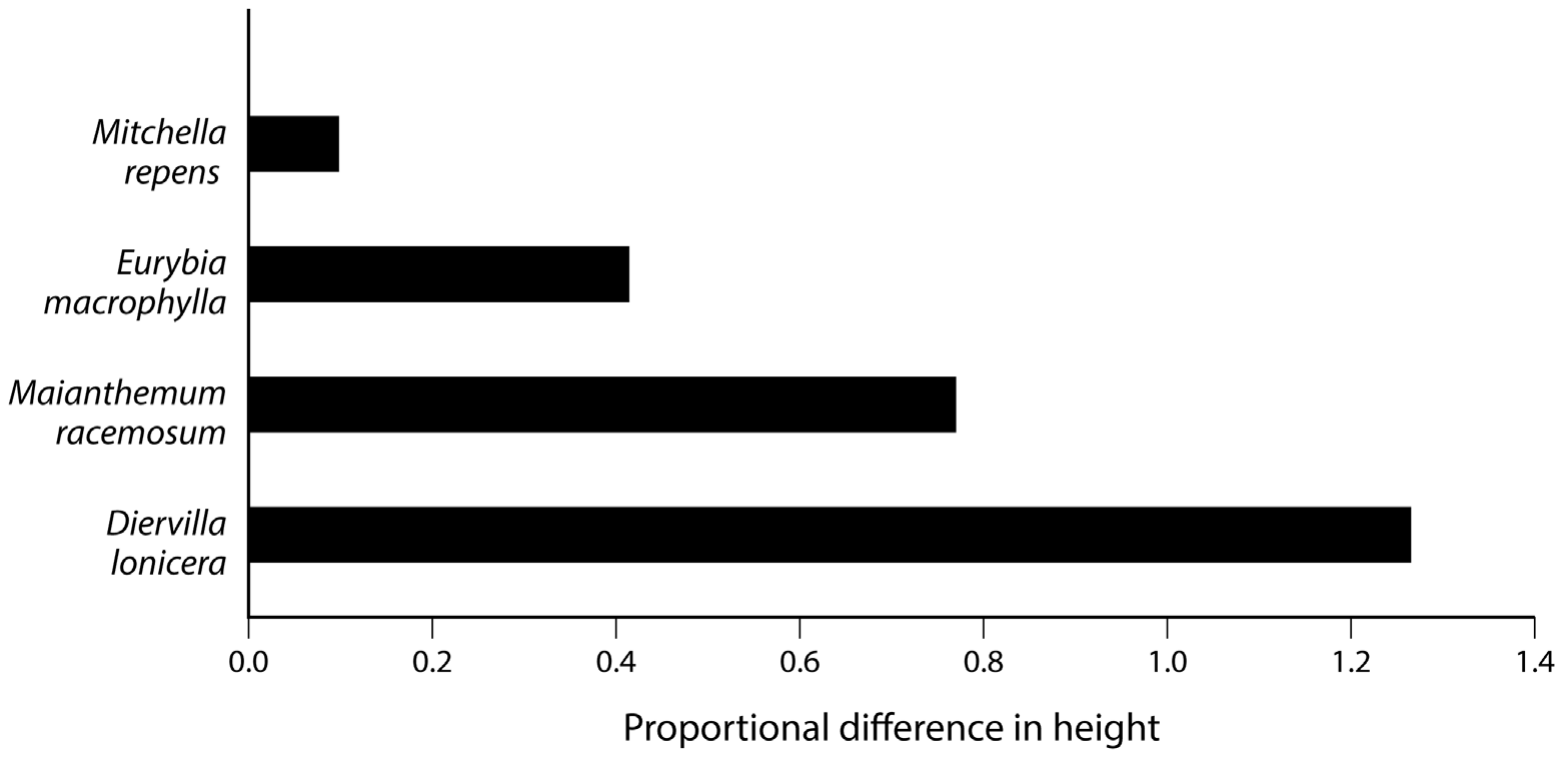
Effects of deer on plant height. Bars compare the mean maximum leaf heights of four focal species between the exclosure (protected from deer) and browsed plots (accessible by deer) as measured by the logarithm of their proportional differences. Depicted species differ significantly between treatments in a mixed model ANOVA using exclosure as the main effect and site as a random effect. *Mitchella*, *Eurybia*, and *Diervilla* have declined in the region over the past 50 years.

Across all exclosure and browsed plots, none of the 36 focal species showed more reproductive individuals or taller leaves in the plots accessible to deer. Eleven species had more reproductive individuals inside the exclosures. Six of these preferentially browsed species have declined across the region over the last half of the 20^th^ century (*Diervilla lonicera*, *Trillium grandiflorum*, *Uvularia grandiflora*, *Viola* spp., all p<0.0001; plus *Mitchella repens*, p = 0.02, and *Streptopus lanceolatus*, p = 0.001; Fig. 2). However, five of these species (*Anemone quinquefolia*, *Maianthemum canadense*, *Maianthemum racemosum*, *Trientalis borealis*, and *Veronica officinalis*) actually increased since the 1950s (all differences p<0.0001). Four species had greater maximum leaf heights inside the fence including one regional increaser (*Maianthemum racemosum*, p<0.0001) and three regional decreasers (*Eurybia macrophylla* and *Mitchella repens*, both p<0.0001, and *Diervilla lonicera*, p = 0.006; Table 4 and Fig. 3). Thus, we see that deer tend to reduce plant height and/or reproduction even in species that have increased in abundance in the presence of deer.

**Table 4:** Results from ANOVAs of maximum leaf heights in each of four focal species.

Species	F	d.f.	P-value
*Diervilla lonicera*	3.5	7, 32	**0.006**
Site	4.6	3	**0.009**
Exclosure	8.0	1	**0.008**
Interaction	2.2	3	0.11
*Eurybia macrophylla*	7.2	7, 32	**<0.0001**
Site	14.1	3	**<0.0001**
Exclosure	4.7	1	**0.038**
Interaction	1.2	3	0.33
*Maianthemum racemosum*	3.6	7, 32	**0.006**
Site	3.6	3	**0.025**
Exclosure	14.2	1	**0.0007**
Interaction	1.64	3	0.20
Michella repens	16.8	7, 32	**<0.0001**
Site	32.1	3	**<0.0001**
Exclosure	9.2	1	**0.005**
Interaction	14.6	3	**0.013**

Table shows F-values for variables predicting maximum leaf height with site, exclosure and their interaction as factors.

### Do responses to deer reflect long-term regional shifts in abundance?

**Figure 4:**
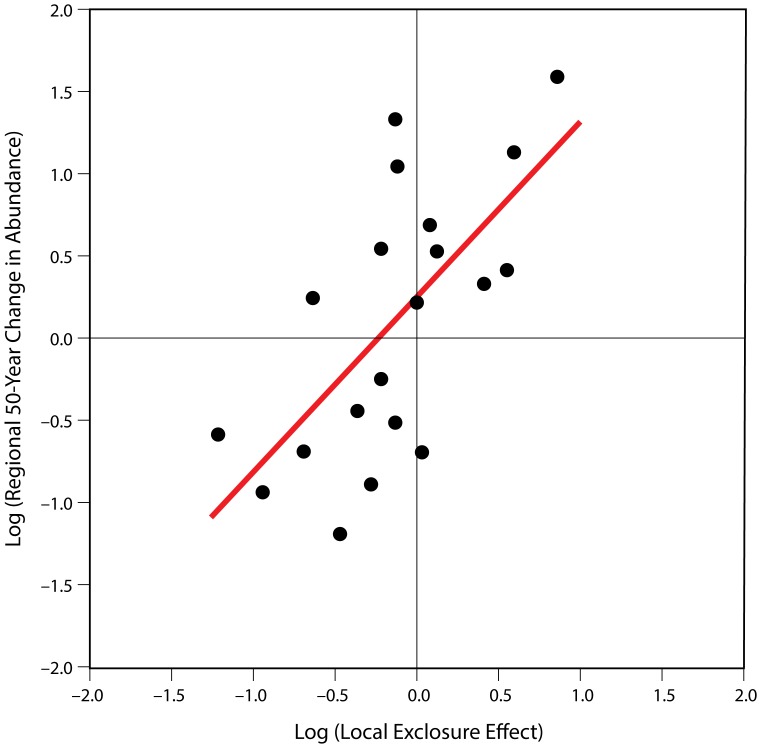
Local deer effects predict long-term regional changes in abundance. The graph shows species' proportional changes in regional abundance over the late 20^th^ century (1950s–2000s) plotted as a function of the deer exclosure effect (the proportional differences in abundance due to the exclosure). Points represent the 20 species that occurred with adequate frequency in both data sets. Slope  = 1.07, Adj. r^2^ = 0.41, F = 14.4, p = 0.0013.

The proportional differences in abundance that we observed within vs. outside the exclosures mirrored observed long-term regional changes in abundance over the past 50 years (Fig. 4). For a subset of 20 focal species, we detected a strong correlation (R^2^ = 0.41) between measures of local deer effects and long-term regional changes in plant abundance. Since these 20 focal species are mostly common, the regression likely underestimates the overall historical effect of deer on these plant communities (see Methods).

## Discussion

Ecologists rarely have the opportunity to link results from rigorous, short-term experiments to longer-term and broader-scale studies. Here, we used results from experimental exclosures to isolate the effects of a single ecological factor – herbivory by white-tailed deer – and examine whether the impacts demonstrated in these experiments corresponded to half-century shifts in forest plant community composition across the broad region of the upper Midwest, USA. In this case, we took advantage of existing exclosures and an unusually detailed set of 1950s baseline data and 2000s resurvey data. Plant responses to deer exclusion largely match observed long-term regional shifts in abundance. The close correspondence between the exclosure results and long-term regional changes in species abundance suggest that deer have indeed substantially altered plant community dynamics over the region for decades. Our study also illustrates the value of linking experimental to observational data at divergent temporal and spatial scales.

Forest structural diversity, plant height, the abundance of several herbaceous species and tree seedlings, and the frequency of flowering and fruiting all declined outside the exclosures. From this, we infer that white-tailed deer have driven parallel changes in forest community structure and composition throughout the region. A landscape once dominated by a diverse set of forbs, shrubs, and regenerating tree seedlings is now increasingly dominated by ferns, grasses, *Carex pensylvanica*, and exotics [32]. Of the 31 most abundant species in our study, almost half showed strong differences in abundance between in- and outside the exclosures. The species that benefitted from protection from deer also tend to be the species that have declined since 1950 while many that thrive in the presence of deer have increased in abundance since 1950 [33].

Given the potential complexity of plant communities and long-term ecological change, it is remarkable to see such a close relationship between deer-mediated differences in abundance (across the exclosure fences) and regional trends in abundance over the past 50 years (Fig. 4). Single factors rarely account for complex long-term changes in community composition and structure. The results presented here, however, support such a conclusion and the predictions made by Alverson & Waller [56], Rooney & Waller [37], and Côté et al. [12] that deer are acting as a “keystone herbivore” to drive long-term ecological changes in these forests.

The particular value of these exclosure results lies in what they tell us about how the impacts of deer differ among species. We did not detect any overall difference in species richness or diversity between the exclosure and browsed plots, suggesting that these aggregate metrics provide poor indicators for deer impacts (in agreement with Royo et al. [41]). This result reflects the fact that roughly similar numbers of species benefited or suffered from the effects of deer. Several species proved to be highly vulnerable to deer as reflected by their declines in, or disappearance from, the browsed plots. Many of these have also been in regional decline over the past 50 years (Table 2). These are native species that tend to be biotically pollinated and dispersed. In contrast, species that occurred more abundantly outside the exclosures are mostly abiotically pollinated and dispersed ferns and graminoids – matching traits and species that have generally increased across the region. Declines in biotically pollinated and dispersed species might reflect direct browsing or the dramatic effects that abundant deer can have on forest animal communities, e.g., by altering the abundance and quality of plant food resources and habitat available to pollinators and songbirds [29], [57], [58], [64].

Several taxa did not differ significantly in abundance between exclosure and browsed plots. Three of these experienced historical declines (*Aralia nudicaulis*, *Eurybia macrophylla*, and *Viola* spp.) while seven (*Arisaema triphyllum*, *Carex* spp., *Dryopteris intermedia*, *Maianthemum canadensis*, *Maianthemum racemosum*, *Trientalis borealis* and *Veronica officinalis*) have increased [33]. Our inability to detect differences in abundance across the fence in these exclosures could reflect low statistical power. As species consumed by deer become rare on the landscape, they may become too infrequent to be included in statistical analyses or so sparse that we have low power to detect differences in abundance. For example, *Taxus canadensis* (Canada yew), is heavily browsed by deer wherever it is encountered. We only observed *Taxus* seven times - all within exclosures. Within the 992 quadrats sampled, six declining species (*Circaea alpina*, *Clintonia borealis*, *Cornus canadensis*, *Osmorhiza claytonii*, *Uvularia sessifolia*, and *Waldsteinia fragarioides*) occurred in fewer than 2% of the quadrats. Apparent lack of exclosure effects may also reflect occasions where populations of sensitive species within the fence have not yet had the opportunity to recolonize or recover from decades of browsing. We could not include rarer species in our analyses but encourage other researchers to devise methods to determine just how sparse plant populations of various species can become before they lose their ability to rebound from heavy herbivory.

### Summary

Forests in the upper Midwest have experienced strong shifts in community composition with declines in herb, shrub, and tree species diversity and vertical structure with cascading impacts on other species [12], [59]. Our results comparing broad regional changes since the 1950s to exclosure effects on plant abundance suggest that many of the historical changes observed reflect the effects of deer. Combining experiments with observational data reinforces the inferences we can make from both approaches allowing us to conclude that deer are having heavy impacts on these plant communities. Similar efforts elsewhere will eventually allow managers to recognize thresholds at which deer populations still allow plant populations and communities to retain their resiliency and ability to recover [60], [61].
