# Correction: Long-Term Regional Shifts in Plant Community Composition Are Largely Explained by Local Deer Impact Experiments

**DOI:** 10.1371/journal.pone.0185037

**Published:** 2017-09-13

**Authors:** Katie Frerker, Autumn Sabo, Donald Waller

Upon review of the dataset used in this manuscript, the authors discovered that the abundance data used in two analyses were inadvertently scrambled, resulting in inflated abundance values. The erroneous numbers affect two of the analyses in the paper, however the initial findings still hold. The affected content is corrected below.

In the Abstract, the fourth and fifth sentences should read: “Among common species, 12 were more abundant outside the exclosures, 14 were commoner inside, and 66 had similar abundances in- and outside. Deer herbivory greatly increased the abundance of graminoids and nearly doubled the abundance of exotic plants.” The ninth sentence should read: “The effects of herbivory by white-tailed deer account for many of the long-term regional shifts observed in species’ abundances (R^2^ = 0.25).”

In the “Data on 50-year shifts in abundance” section, the first three sentences should read: “To test whether the differences in species abundance that we observed in- vs. outside the exclosures were related to the long-term (1950s to 2001) regional shifts in abundance measured by Wiegmann and Waller [33] over 62 sites, we first calculated the change in abundance across the fence line as the arithmetic difference of each taxon’s frequency in the control browsed plots minus its frequency inside the exclosure plots. We then calculated each taxon’s change in abundance over the last 50 years as the arithmetic difference between its frequency in 2000 minus its frequency in the 1950s. We excluded rare species that occurred less than 5 times in both data sets, leaving 53 species for the analysis.”

In the “Data analyses” section, the last sentence of the first paragraph reads: “We restricted these tests to species that occurred in at least 50 quadrats overall and had expected frequencies greater than five in each treatment.” Because of the lower abundances in the corrected dataset, the authors no longer put the 50 quadrat restriction on the analysis and the text should now read: “We restricted these tests to species that had expected frequencies of greater than five in each treatment.”

Because the authors no longer use the 50 quadrat restriction, the first sentence of the “Species’ responses to deer” section should read: “Of the 256 species encountered in the exclosure and browsed plots, 97 occurred commonly enough (expected value of 5) to analyze differences in abundance in- and outside the fences using χ^2^ tests.”

Some of the results have therefore changed, and the remainder of the “Species’ responses to deer” section should read: “Twelve species were more abundant in browsed plots while fourteen were more abundant in exclosures (Table 2). The other 66 species did not differ in abundance between treatments. Species that increased in the presence of deer included three native forbs (*Coptis trifolia*: 4.5x, *Rubus pubescens*: 4x, and *Symphyotrichum lateriflorum*: 2x), two non-native forbs (*Hieracium aurantiacum*: 4.5x and *Lapsana communis*: 2x), a fern (*Pteridium aquilinum*: 7x), two native (*Schizachne purpurascens*: 2.8x and *Carex pensylvanica*: 2.5x) and one non-native graminoids (*Poa compresa*: 5.5x), and three woody species (*Ostrya virginiana*: 1.6x, *Fraxinus nigra*: 3x, and *Corylus cornuta*: 10x). Overall, exotic species were 1.5x as abundant in the browsed (control) vs. exclosure plots (336 vs. 238 occurrences, *χ*^2^ = 20.04, p<0.0001). Conversely, six woody species, one native graminoid, and seven forbs (including four declining focal species) were more abundant inside the exclosures. These forbs were *Trillium grandiflorum*, *Aralia nudicaulis*, *Hieracium piloselloides*, *Maianthemum racemosum*, *Prunella vulgaris*, *Uvularia grandiflora* and *Viola pubescens*. Seedlings of *Thuja occidentalis* and *Tsuga canadensis* were much scarcer outside the exclosures. The shrubs *Cornus rugosa* and *Diervilla lonicera* also occurred 100–200 times more often within the exclosures than in browsed plots (mean intercepts of 328.3 vs. 15.5 cm and 199.7 vs. 2.0 cm, respectively, Table 3). Combining taxa to genus, *Poaceae* spp. and exotic *Lonicera* spp. were more abundant in the control / browsed plots. *Viola* spp. were more abundant in exclosures. In sum, graminoids and exotic species thrived in the presence of deer while native forbs, several shrubs and tree seedlings all tended to decline.”

**Table 2:** Differences in species abundances in- and outside deer exclosures.

	Abundance		
Species	Control	Exclosure	Chi-square value
**More Abundant in Control:**			
*Carex pensylvanica*	22	9	5.45 *
*Coptis trifolia*	14	3	7.12 **
*Corylus cornuta*	10	1	7.36 **
*Fraxinus nigra*	19	6	6.76 **
*Hieracium aurantiacum*	9	2	4.45 *
*Lapsana communis*	44	20	9.00 **
*Lonicera* spp. (exotic)	12	4	4.00 *
*Ostrya virginiana*	50	32	3.95 *
*Poa compresa*	11	2	6.23 *
*Poaceae* sp.	37	8	18.69 ***
*Poaceae* spp.	271	195	12.39 ***
*Pteridium aquilinum*	14	2	9.00 **
*Rubus pubescens*	16	4	7.20 **
*Schizachne purpurascens*	14	5	4.26 *
*Symphyotrichum lateriflorum*	30	15	5.00 *
**More Abundant in Exclosure:**			
*Aralia nudicaulis*	21	40	5.92 *
*Brachyelytrum erectum*	17	31	4.08 *
*Cornus rugosa*	7	32	16.03 ***
*Diervilla lonicera*	11	50	24.93 ***
*Hieracium piloselloides*	6	18	6.00 *
*Maianthemum racemosum*	28	46	4.38 *
*Prunella vulgaris*	1	9	6.40 *
*Symphoricarpos albus*	1	9	6.40 *
*Thuja occidentalis*	6	43	27.94 ***
*Trillium grandiflorum*	23	46	7.67 **
*Tsuga canadensis*	25	49	7.78 **
*Uvularia grandiflora*	6	30	16.00 ***
*Viburnum acerifolium*	3	12	5.40 *
*Viola pubescens*	24	52	10.32 **
*Viola* spp.	90	138	10.11 **

Abundance values reflect the incidence of species across quadrats in the control and exclosure plots. We judge whether species are more abundant in or outside exclosures from the *χ*^2^ and significance values for the differences observed. For the taxa listed, “sp.” refers to members of the family or genus that could not be identified to species level, “spp.” refers to all species within the family or genus, grouped together for analysis.

***p<0.001

**p<0.01

*p<0.05.

Table 2 should be changed to reflect the updated findings. Please see the corrected Table 2 here.

In the “Do responses to deer reflect long-term regional shifts in abundance?” section, the second and third sentences should read: “For the subset of 53 focal species, estimated local deer effects and long-term regional changes in abundance are correlated (R^2^ = 0.25). Since these 53 species are generally common, the regression may underestimate the historical effects of deer on the whole plant community.”

Fig 4 should be changed to reflect the updated findings. Please see the corrected Fig 4 here.

**Fig 4:**
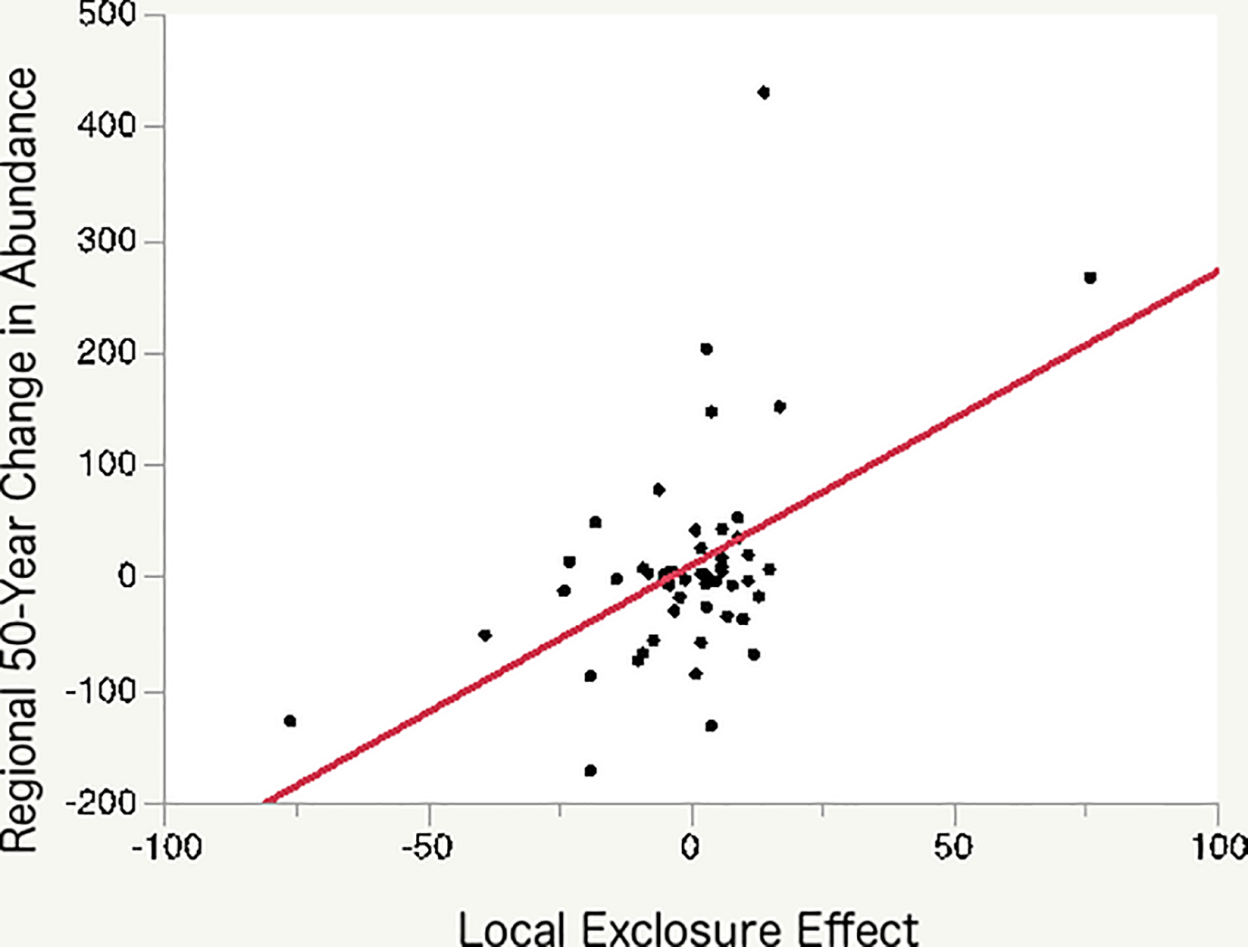
Local deer effects predict long-term regional changes in abundance. The graph shows species' proportional changes in regional abundance over the late 20^th^ century (1950s–2000s) plotted as a function of the deer exclosure effect (the proportional differences in abundance due to the exclosure). Points represent the 53 species that occurred with adequate frequency in both data sets. Slope = 2.6, Adj. r^2^ = 0.25, F = 18.1, p < 0.0001.

In the Discussion section, the third and fourth sentences of the second paragraph should read: “A landscape once dominated by a diverse set of forbs, shrubs, and regenerating tree seedlings is now increasingly dominated by grasses, *Carex pensylvanica*, and exotics [32]. Twenty six of the 92 most abundant species in our study (28%) show strong differences in abundance between in- and outside the exclosures.” The seventh sentence of the fourth paragraph should read: “In contrast, species that occurred more abundantly outside the exclosures are mostly abiotically pollinated and dispersed taxa like ferns and graminoids—matching traits and species that have generally increased across the region.” The second sentence of the fifth paragraph should read: “Six of these experienced historical declines (*Eurybia macrophylla*, *Fragaria virginiana*, *Huperzia lucidula*, *Mitchella repens*, *Rubus parviflorus*, and *Streptopus lanceolatus*) while nine (*Arisaema triphyllum*, *Anemone quinquefolia*, *Athyrium filix-femina*, *Dryopteris intermedia*, *Maianthemum canadense*, *Oryzopsis asperifolia*, *Poa nemoralis*, *Trientalis borealis*, and *Veronica officinalis*) have increased [33].” The sixth and seventh sentences of the fifth paragraph should read: “We only observed *Taxus* eight times–all within exclosures. Within the 992 quadrats sampled, six declining species (*Circaea alpina*, *Clintonia borealis*, *Cornus canadensis*, *Galium aparine*, *Uvularia sessifolia*, and *Waldsteinia fragarioides*) occurred in fewer than 2% of the quadrats.”
